# Can development alone save lives? trends and forecasts of congenital heart anomalies–attributable premature mortality in ASEAN

**DOI:** 10.3389/fcvm.2026.1698307

**Published:** 2026-04-21

**Authors:** Muhammad Iqhrammullah, Derren D. C. H. Rampengan, Starry H. Rampengan, Radityo Prakoso, Teuku Andi Syahputra, Iqbal Farhan Sayudo, Shakira Amirah, Ayers Gilberth Ivano Kalaij, Puan Faiza Nurianada, Muhammad Habiburrahman

**Affiliations:** 1 Postgraduate Program of Public Health, Universitas Muhammadiyah Aceh, Banda Aceh, Indonesia; 2 Faculty of Medicine, Universitas Sam Ratulangi, Manado, Indonesia; 3 Division of Interventional Cardiology, Department of Cardiology and Vascular Medicine, R.D. Kandou Central General Hospital, Universitas Sam Ratulangi, Manado, Indonesia; 4 Division of Paediatric Cardiology and Congenital Heart Disease, Department of Cardiology and Vascular Medicine, National Cardiovascular Centre Harapan Kita, Universitas Indonesia, Jakarta, Indonesia; 5 Department of Pediatrics, Faculty of Medicine, Universitas Syiah Kuala, Banda Aceh, Indonesia; 6 Medical Research Unit, Universitas Syiah Kuala, Banda Aceh, Indonesia; 7 Faculty of Medicine, Universitas Indonesia, Jakarta, Indonesia; 8 Faculty of Medicine, Universitas Jenderal Soedirman, Banyumas, Indonesia; 9 Faculty of Medicine, Imperial College London, London, United Kingdom

**Keywords:** ASEAN, burden of cardiovascular diseases, congenital heart anomalies, socio-demographic index, years of life lost

## Abstract

**Background:**

Congenital heart anomalies (CHAs) are a leading cause of premature childhood mortality and contribute substantially to years of life lost (YLL). Despite overall improvements in child survival in Southeast Asia, progress in reducing CHA-attributed mortality has been uneven. Examining how trends in CHA-related YLL align with socioeconomic development is critical for informing targeted health system investments. This study assesses trends and projections of CHA-attributed premature mortality in ASEAN countries from 2000 to 2030 in relation to development levels.

**Methods:**

Data from the Global Burden of Disease Study 2021 were used to extract CHA-attributed YLL rates, including all-age and age-stratified estimates (<5, 5–9, and 10–19 years), alongside Socio-demographic Index (SDI) values from 2000 to 2021. Estimated Annual Percentage Change (EAPC) for YLL and SDI was calculated using log-linear regression, and an efficiency ratio quantified YLL reduction per unit SDI gain. Forecasts up to 2030 were generated using ARIMA and ARIMAX models applied to the natural logarithm–transformed outcome values, with SDI trajectories stratified by recent growth patterns.

**Results:**

From 2000 to 2021, all ASEAN countries showed declining CHA-attributed YLL. Singapore achieved the steepest decline (from 104.27 to 26.09 per 100,000) and highest efficiency relative to SDI gains. Children under five consistently bore the greatest burden, with 2021 YLLs ranging from 319 per 100,000 in Singapore to 7,435 per 100,000 in Lao PDR, a more than 23-fold disparity. Under SDI-adjusted projections to 2030, Cambodia, Lao PDR, Myanmar, and Timor Leste were projected to achieve moderate reductions of approximately 16–29% compared with 2021, although their absolute YLL levels remained high (YLLs > 600 per 100,000). The estimated trends in Singapore and Brunei Darussalam exceeded those expected under the SDI-adjusted projections, with projected YLLs of 17.35 and 131.67 per 100,000 population, respectively.

**Conclusion:**

CHA-attributed premature mortality is largely preventable but not assured by development alone. Greater YLL reductions occur when investment in pediatric cardiac care keep pace with socioeconomic progress, highlighting the need for targeted interventions in countries with rising SDI but limited mortality reduction.

## 1 Introduction

Congenital heart anomalies (CHAs) are among the most common congenital defects, affecting approximately 1% of live births globally and ranking as the leading cause of congenitally related child mortality (1, 2). In 2021, an estimated 217,000 global deaths among individuals under 20 were attributed to CHAs, and CHAs remained the leading non-communicable cause of pediatric death (3). Alarmingly, children under 5 years bear the highest burden, with mortality rates declined by over 70% in high-SDI regions but only by less than 20% in low-SDI regions between 1990 and 2021 (4, 5).

Previous burden of disease reports rely on Disability-Adjusted Life Years (DALYs) (4, 6, 7). YLL-based reporting, on the other hand, is relevant for CHAs due to the fact that premature death constitutes the majority of the disease burden (8). As reported previously, children with CHAs are prone to failure to thrive, often due to rapid clinical decline and limited time spent living with long-term disability (9, 10). A GBD data-based investigation further supports this pattern, demonstrating that over 98% of the total DALY burden for CHAs in children under five is attributable to YLL rather than YLD (7). By isolating YLL, this study aims to highlight the underreported mortality impact of CHAs and draw greater policy attention to preventable early deaths, especially in countries with limited pediatric cardiac capacity. A recently published global burden analysis on CHA corroborated this rationale by showing that, although prevalence remained stable from 1990 to 2021, CHD-related mortality and DALYs in children under five remain disproportionately high in low-SDI countries (6). Unfortunately, previous GBD studies on CHAs did not report YLL estimates separately from DALYs, underscoring the need for focused YLL analyses to better capture the full extent of early-life mortality and to guide more targeted interventions for pediatric cardiac health (4, 6, 7).

The Association of Southeast Asian Nations (ASEAN), home to more than 680 million people, has rapidly advanced in economic development and human capital over the past two decades (11). However, this progress has not been matched by uniform gains in health outcomes (11, 12). Funding for healthcare in ASEAN countries is often centralized through government systems, yet limited resources are allocated to pediatric cardiovascular diseases despite their high mortality burden. In the context of CHAs, substantial disparities remain in mortality rates and health system responses across member countries. In Indonesia, for instance, burden of CHAs is twice higher as compared to non-congenital pediatric heart diseases (13). These inconsistencies challenge the assumption that socioeconomic growth automatically reduces preventable deaths (14, 15). Given ASEAN's increasing integration in health policy and shared aspirations toward Universal Health Coverage and the Sustainable Development Goals, understanding the patterns and projections of premature mortality due to CHAs is crucial for informing targeted interventions (16).

Understanding how national development influences this burden is critical. The Socio-demographic Index (SDI), a composite measure of income, education, and fertility, offers a standardized proxy for development (17). By tracking SDI alongside YLL across ASEAN countries, we can better interpret progress and identify missed opportunities in translating socioeconomic gains into child survival improvements (18). Thus, by using the data from the GBD 2021, the study aimed to assess CHA-attributed YLL trends in ASEAN countries. It also projects future trends through 2030 using time-series models, both with and without SDI integration, to examine how development trajectories might influence future outcomes and whether disparities are likely to persist.

## 2 Methods

### 2.1 Study design and data sources

This was a retrospective modeling study using publicly available data from the GBD Study 2021, provided by the Institute for Health Metrics and Evaluation (IHME) through the Global Health Data Exchange (GHDx) platform (3). The GBD study is a large-scale international collaboration involving over 10,000 researchers from more than 160 countries, aiming to provide standardized estimates of disease burden across time, geography, and population (19).

Annual all-age YLL rates due to CHAs were extracted for 11 ASEAN countries, namely Singapore, Brunei Darussalam, Malaysia, Thailand, Indonesia, Viet Nam, Philippines, Cambodia, Myanmar, Lao People's Democratic Republic (PDR), and Timor Leste. Additionally, SDI values were collected for the same time period (20). Subgroup analysis was also performed by age range categories: <5 years, 5–9 years, and 10–19 years, to explore premature mortality distribution across developmental stages.

### 2.2 YLL and SDI estimation

YLLs were estimated in the GBD framework using cause-specific mortality counts and the GBD reference life expectancy at each age of death (3). GBD 2021 estimated cause-specific mortality and YLLs using over 56,000 unique data sources, including national and sample vital registration systems, verbal autopsies, cancer registries, household surveys, censuses, police records, and surveillance systems (21). To ensure epidemiological consistency and enhance the precision of estimates, the GBD study group employed two modeling frameworks. DisMod-MR 2.1, a Bayesian meta-regression tool, was utilized to harmonize key parameters such as incidence, prevalence, and mortality. Additionally, Spatiotemporal Gaussian Process Regression (ST-GPR) was applied to account for spatial and temporal heterogeneity by smoothing trends and leveraging information across neighboring regions and time points (19). The SDI, a composite indicator ranging from 0 to 1, incorporates income per capita, mean years of education, and fertility rate, serving as a proxy for national development. It reflects a country's capacity to provide health services and mitigate disease burden through socioeconomic advancement. Higher SDI values indicate better socioeconomic conditions, typically associated with stronger health systems and improved child survival rates (20).

### 2.3 Trend estimation and efficiency metrics

To evaluate temporal trends, we calculated the Estimated Annual Percentage Change (EAPC) for both YLL and SDI using the log-linear model (Equation 1):

$$EAPC=100\;\times \;(e^{\beta }\text{-}1)$$ (1)

where *β* is the slope from a linear regression of log-transformed YLL or SDI against calendar year. To assess the efficiency of health improvement relative to development, we calculated the EAPC ratio (Equation 2):

$$Efficiency=\;\frac{EAPC_{YLL}}{EAPC_{SDI}}$$ (2)

A more negative ratio indicates greater YLL reduction per unit of SDI improvement.

### 2.4 Forecasting model

YLL trends were projected to 2030 using two time-series forecasting approaches: an autoregressive integrated moving average model (ARIMA) and an ARIMA model with exogenous regressors (ARIMAX). Both models were applied to the natural logarithm of YLL to stabilize variance and ensure non-negative forecasts after back-transformation. The ARIMA model relied solely on historical YLL observations, whereas the ARIMAX model additionally incorporated the SDI as an external predictor to account for the influence of socioeconomic development on mortality trends.

The general ARIMA model can be expressed as:

$$\phi (B)(1\text{-}B{)}^{d}\ln(YLL_{t})=c+\theta (B)\varepsilon_{t}$$ (3)

where *B* is the backshift operator, *d* denotes the order of differencing, ϕ(B) and θ(B) represent autoregressive and moving-average polynomials, *c* is the drift term, and $\varepsilon_{t}$ is the error term.

For the ARIMAX model, SDI was included as an external regressor:

$$\phi (B)(1-B{)}^{d}\ln(YLL_{t})=c+\beta \;SDI_{t}+\theta (B)\varepsilon_{t}$$ (4)

where β represents the effect of socioeconomic development on YLL trajectories.

Model fitting was conducted using the auto.arima() function from the *forecast* package in R. The optimal model orders (p,d,q) were automatically selected using the Akaike Information Criterion corrected for small samples (AICc). Residual diagnostics were performed to ensure that the fitted models did not exhibit remaining autocorrelation.

To propagate uncertainty from the GBD estimates, a Monte Carlo simulation approach was applied. For each country and year, YLL values were sampled from a log-normal distribution defined by the reported mean and 95% UI. Specifically, the parameters of the distribution were derived as:

$$\mu =\ln(YLL),\sigma =\frac{\ln(YLL_{upper})-\ln(YLL_{lower})}{3.92}$$ (5)

A total of 1000 simulated time series were generated for each country. ARIMA and ARIMAX models were fitted to each simulated series, and the resulting forecasts were summarized using the mean and the 2.5th and 97.5th percentiles of the simulated distribution to produce forecast uncertainty intervals.

Because ARIMAX forecasting requires future SDI values, SDI trajectories were projected based on the EAPC of SDI for each country. Countries were classified into three development-velocity groups according to the empirical distribution of SDI EAPC values across ASEAN countries. Countries with EAPC below the lower observed cluster (EAPC < 0.6) were assigned a near-flat projection representing slow structural development. Countries within the central distribution (0.6 ≤ EAPC < 1.5) followed a linear extrapolation based on the average change observed in the most recent years. Countries within the upper tail of the distribution (EAPC ≥ 1.5) were projected using an exponential growth function derived from the recent compound annual growth rate:

$$SDI_{t+h}=SDI_{t}(1+g{)}^{h}$$ (6)

where *g* represents the estimated annual SDI growth rate.

This classification was derived from the empirical distribution of SDI growth rates within the ASEAN dataset and was used to represent plausible development trajectories rather than universal thresholds. Forecasts were generated until 2030 and subsequently back-transformed to the original YLL scale. All data processing, simulation, and visualization were performed in R version 4.4.0 using RStudio 2024.04.2-764.

### 2.5 Ethical consideration

This study analyzed publicly available, aggregated country-level estimates derived from the GBD 2021 study. No primary data collection was conducted, and no individual-level, identifiable, or private information was accessed. As the analysis relied exclusively on anonymized secondary data in the public domain, formal ethical approval and informed consent were not required, in accordance with institutional and national research ethics guidelines and the ethical principles of the Declaration of Helsinki (2013 revision).

## 3 Results

### 3.1 Trends of CHA-related YLL across ASEAN countries

**Figure 1:**
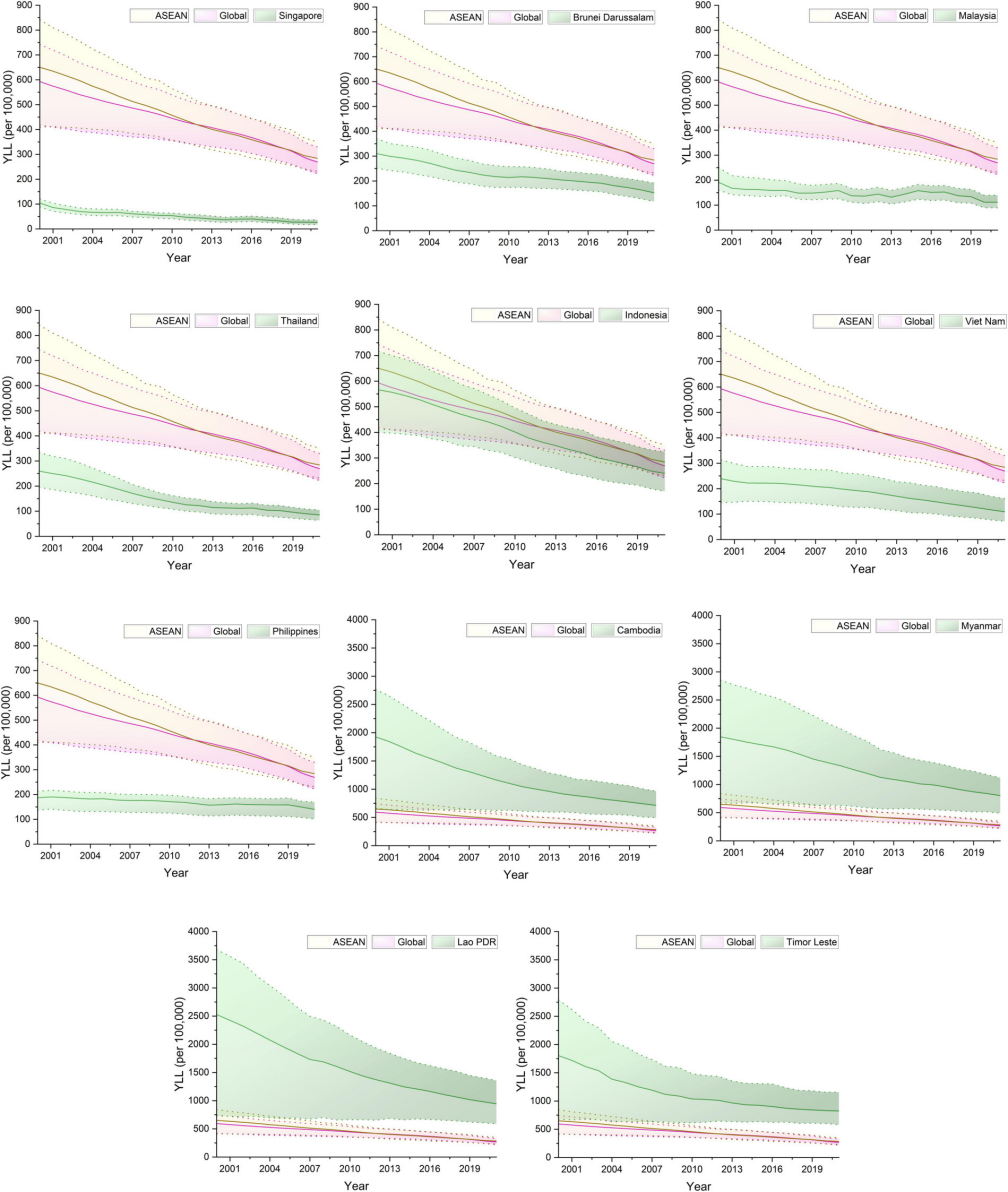
Trends in years of life lost (YLL) from congenital heart anomalies across ASEAN countries compared to regional and global benchmarks, 2000–2019.

From 2000 to 2021, all ASEAN countries experienced a decline in all-age YLL due to CHA (Figure 1). However, the extent of progress and alignment with global and regional (ASEAN) averages varied substantially. In 2021, the global average YLL was approximately 270 per 100,000, while the ASEAN average stood at around 283 per 100,000. Only Singapore performed consistently below both benchmarks, with YLL declining from 104 per 100,000 in 2000 to 26 per 100,000 in 2021. Thailand, Brunei Darussalam, Malaysia, The Philippines, and Viet Nam also fell below the global and ASEAN averages by 2021, reaching 85–161 per 100,000. Indonesia saw substantial reductions but remained below the global and ASEAN averages, with YLL of 240 per 100,000 in 2021. Cambodia showed notable improvement, cutting YLL from 1,926 per 100,000 in 2000 to 713 per 100,000 in 2021. Myanmar recorded the YLL of 1846 per 100,000 in 2000, and declined to 804 per 100,000 in 2021. CHA in Timor Leste achieved a slightly lower YLL (1802 per 100,000) than that in Myanmar in 2021. Lao PDR consistently recorded the highest burdens, with YLLs of 2528 per 100,000 in 2000 and 944 per 100,000 in 2021.

### 3.2 CHA-related YLL in specific age groups

**Figure 2:**
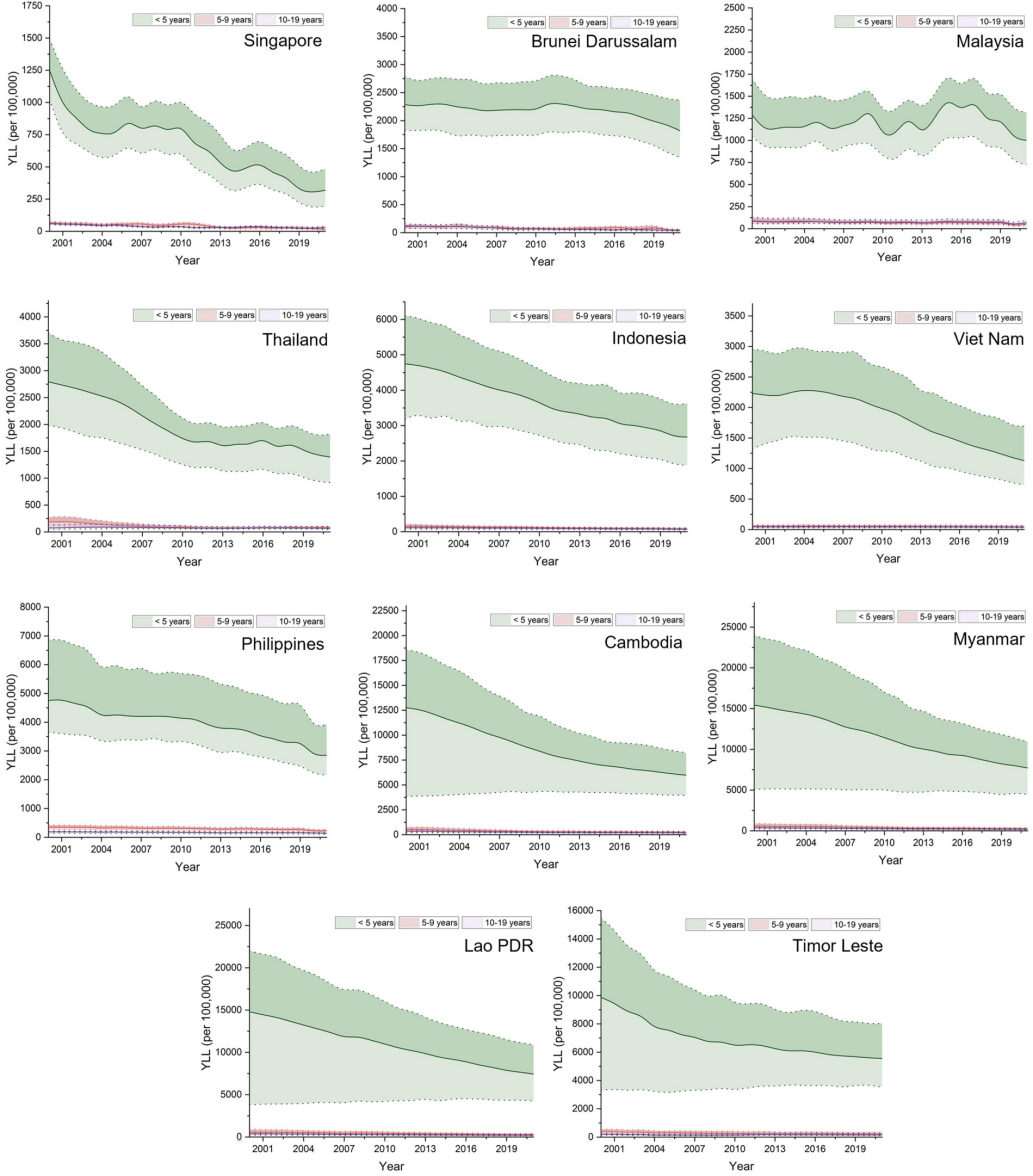
Trends in years of life lost (YLL) due to congenital heart anomalies across age groups (<5, 5–9, and 10–19 years) in ASEAN countries, 2000–2019.

Across ASEAN countries, children under five years of age (<5 years) consistently bore the highest YLL burden from CHA (Figure 2). However, the magnitude and reduction trajectory varied widely. Singapore showed a remarkable and sustained decline in <5 YLL, falling from 1,247 per 100,000 in 2000 to under 400 by 2021, with this group converging toward older age strata by the end of the period. Brunei Darussalam also exhibited meaningful suppression in premature YLL, decreasing steadily from approximately 2,285 to 1,820 per 100,000. Viet Nam followed a different yet distinct trajectory: <5 YLL levels remained relatively stable around 2,200 per 100,000 from 2000 to 2007, before a clear inflection point in 2008 marked the beginning of a consistent decline, reaching 1,128 per 100,000 by 2021.

Thailand and Malaysia achieved moderate reductions in <5 YLL—by 2021, although fluctuations remained in Malaysia. Notably, Thailand's < 5 YLL declined from around 2,794 to around 1,394 per 100,000. In contrast, Cambodia, Lao PDR, and Timor Leste still exhibited extremely high <5 YLL throughout the period. In 2021, Lao PDR recorded the highest burden, with <5 YLL exceeding 7,000 per 100,000, and Cambodia and Timor Leste followed closely with >5,000 per 100,000. In these countries, <5 YLL was over 20 times higher than that in older children. Indonesia, Myanmar, and the Philippines showed steady yet insufficient progress. For instance, Indonesia's < 5 YLL dropped from 4,745 to 2,677 per 100,000, yet remained substantially higher than the 5–9 and 10–19 age groups (Figure 2).

### 3.3 EAPC analysis and YLL-to-SDI gains

Over the two-decade period from 2000 to 2021, improvements in human development—as measured by increases in the SDI—were associated with marked reductions in YLL due to CHAs across ASEAN countries (Table 1). Countries with higher baseline SDI and greater gains over time, such as Singapore (SDI: 0.77 to 0.86), achieved the most substantial decline in YLL, decreasing from 104.27 to 26.09 per 100,000 (EAPC: −5.74%). Notably, Singapore demonstrated the highest relative efficiency, indicating a greater proportional reduction in YLL relative to proportional gains in SDI. Thailand also showed high relative efficiency, with YLL declining more rapidly than SDI increased (from 259.71 to 85.08 per 100,000; SDI: 0.58 to 0.68). In contrast, countries with lower baseline SDI, namely Lao PDR, Cambodia, and Myanmar, also experienced meaningful absolute reductions, but exhibited lower relative efficiency, with proportional YLL reductions lagging behind proportional SDI gains. For example, despite having one of the largest relative increases in SDI (0.32 to 0.49), Lao PDR showed a comparatively smaller proportional decline in YLL.

**Table 1:** Changes in SDI, all-age YLL, and EAPC due to CHAs across ASEAN countries, 2000–2021.

Country	SDI		YLL (95% UI)		EAPC		
	2000	2021	2000	2021	SDI	YLL	YLL/SDI
Singapore	0.77	0.86	104.27 (120.98, 88.81)	26.09 (36.76, 17.43)	0.53	−5.74	−10.74
Brunei Darussalam	0.72	0.81	310.15 (370.25, 251.70)	152.27 (191.33, 116.84)	0.53	−3.00	−5.68
Malaysia	0.63	0.74	191.21 (250.76, 160.77)	111.43 (139.89, 89.02)	0.77	−1.49	−1.93
Thailand	0.58	0.68	259.71 (334.01, 195.18)	85.08 (105.30, 63.36)	0.77	−5.29	−6.89
Indonesia	0.53	0.66	566.1 (716.96, 398.94)	240.16 (324.38, 169.81)	1.05	−4.16	−3.94
Viet Nam	0.49	0.63	239.07 (312.74, 145.93)	108.91, (160.73, 71.72)	1.17	−3.50	−2.99
Philippines	0.54	0.65	752.58 (1051.91, 599.72)	359.20 (464.48, 287.57)	0.88	−3.17	−3.60
Cambodia	0.34	0.47	1925.68 (2764.65, 661.30)	712.72 (965.25, 496.68)	1.65	−4.69	−2.85
Myanmar	0.37	0.53	1846.10 (2853.76, 675.33)	803.64 (1120.48, 491.99)	1.78	−4.07	−2.28
Lao PDR	0.32	0.49	2527.88 (3680.27, 729.93)	943.86 (1357.53, 589.45)	2.00	−4.66	−2.33
Timor Leste	0.34	0.44	1802.87 (2790.60, 675.288)	823.68 (1157.77, 581.815)	1.33	−3.64	−2.75

### 3.4 Projections of CHA-attributed YLL to 2030

**Figure 3:**
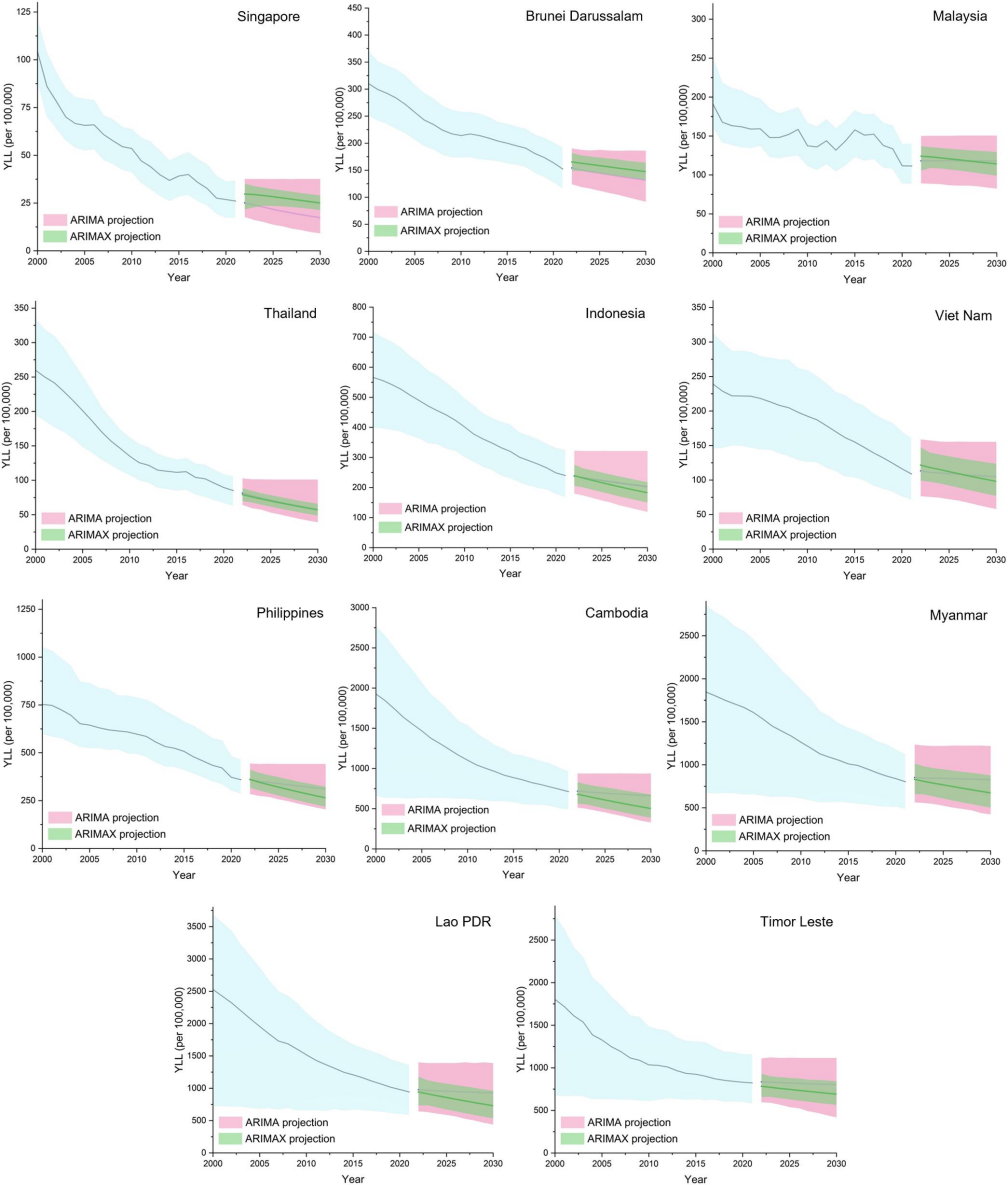
Forecasted years of life lost (YLL) due to congenital heart anomalies in ASEAN countries from 2022 to 2030 using ARIMA (light blue) and ARIMAX (green) models. The ARIMAX model incorporates the SDI to reflect the potential influence of human development on future mortality trends. Shaded areas indicate 95% uncertainty intervals (UI).

The projected YLLs due to congenital heart anomalies across ASEAN countries under ARIMA and ARIMAX models are presented in Figure 3. Forecasting analyses comparing ARIMA (Model 1) and ARIMAX (Model 2) revealed differing trends in projected YLL across ASEAN countries (Table 2). By 2030, as compared to ARIMA, ARIMAX projected markedly lower YLL values in countries such as Cambodia (661.50 vs. 501.97), Lao PDR (930.25 vs. 729.79), and Timor Leste (801.64 vs. 691.04).

**Table 2:** Forecasted YLL due to CHAs per 100,000 population in ASEAN countries for 2025 and 2030.

Country	Forecasted YLL (95% CI)			
	2025		2030	
	Model 1	Model 2	Model 1	Model 2
Singapore	21.74 (13.73, 37.52)	28.33 (23.50, 32.32)	17.35 (9.21, 37.62)	25.06 (21.32, 29.04)
Brunei Darussalam	144.53 (112.06, 187.68)	158.39 (144.24, 173.56)	131.67 (91.76, 186.17)	147.44 (132.40, 164.18)
Malaysia	118.58 (86.74, 150.15)	120.66 (107.02, 133.61)	117.79 (82.40, 150.49)	114.09 (98.91, 129.38)
Thailand	71.24 (53.10, 101.04)	70.04 (61.65, 78.42)	57.61 (39.02, 101.04)	57.18 (48.75, 65.93)
Indonesia	223.60 (157.27, 321.42)	216.13 (185.08, 249.75)	202.99 (119.13, 321.28)	182.65 (150.40, 217.93)
Viet Nam	109.13 (72.36, 155.27)	112.18 (92.16, 134.49)	104.81 (58.11, 155.27)	98.09 (77.14, 123.63)
Philippines	339.47 (256.91, 441.60)	320.60 (276.44, 372.90)	311.47 (204.64, 441.60)	264.63 (217.97, 323.02)
Cambodia	692.61 (455.65, 938.53)	608.50 (499.71, 765.70)	661.50 (330.08, 938.53)	501.97 (387.48, 677.03)
Myanmar	842.35 (524.98, 1218.22)	767.31 (607.75, 948.10)	828.40 (424.65, 1218.22)	672.95 (502.54, 878.33)
Lao PDR	955.41 (588.90, 1389.07)	857.45 (671.90, 1073.20)	930.25 (438.87, 1389.07)	729.79 (535.05, 963.99)
Timor Leste	822.58 (539.54, 1118.14)	746.78 (629.02, 881.59)	801.64 (418.54, 1115.64)	691.04 (564.47, 843.21)

Model 1 (ARIMA) projections are based solely on historical YLL trends, while Model 2 (ARIMAX) incorporates SDI as an external predictor.

In countries with more stable or high SDI levels, SDI-adjusted projections slightly exceeded or closely matched with that of trend-based. In Brunei Darussalam, SDI-adjusted projection in 2030 estimated YLL at 147.44 per 100,000 compared to 131.67 that of trend-based. Similarly, in Indonesia, both models projected persistent burden, with 2030 YLL estimates of 182.65 (SDI-adjusted) and 202.99 per 100,000 (trend-based). Thailand has the closest agreement between the SDI-adjusted (57.18 per 100,000) and trend-based projections (57.61 per 100,000). Singapore as a high-SDI country was projected to maintain the lowest burden of CHAs in 2030 among ASEAN countries with 17.35 per 100,000 in trend-based projection and 25.06 per 100,000 in SDI-adjusted projection. A widening gap between trend-based and SDI-adjusted projections was observed in countries within the lowest SDI tier, as well as in Indonesia and the Philippines.

## 4 Discussion

This present study provides compelling evidence that premature death due to CHAs in Southeast Asia can be reduced with appropriate development and health system strategies. However, because the GBD framework aggregates multiple congenital cardiac conditions into a single cause category, the observed reductions likely reflect improvements across a heterogeneous group of lesions, with greater survival gains expected for simpler defects such as ventricular septal defects (VSD), atrial septal defects (ASD), and patent ductus arteriosus (PDA) compared with more complex lesions such as transposition of the great arteries or single-ventricle physiology. Most countries experienced greater YLL reductions when SDI was introduced into the forecasting model, suggesting a tight coupling between development gains and health system performance. Singapore is a good example of how reductions in YLL can surpass levels predicted by socioeconomic development alone when strong health system capacity and specialized congenital cardiac care are in place. Despite already achieving low YLL in 2000, Singapore continued to improve. In Singapore, successive public health strategies—from early tobacco control to the establishment of the Health Promotion Board, development of the Singapore-modified Framingham Risk Score (SG-FRS), and the nationwide Healthier SG initiative (22, 23). These are coupled with evidence-based interventions such as multicomponent primary care models and investments in longitudinal research (24). These sustained efforts have translated into projected SDG attainment scores above 93 by 2030, with top-tier performance in maternal and child health (MCH), NCDs, UHC, and environmental health (25).

Moreover, one key factor underlying this performance is the highly integrated congenital cardiac care pathway in Singapore, which includes routine prenatal screening, early neonatal detection through pulse oximetry and echocardiography, rapid referral to specialized pediatric cardiac centers, and timely access to corrective or palliative surgery (26–28). These coordinated systems enable early intervention even for complex CHAs, substantially reducing perioperative mortality and long-term complications that would otherwise contribute to YLL. Notably, Singapore recorded the highest efficiency in YLL reduction per SDI point gained (–10.74). As suggested in previous studies, smart governance and targeted cardiac care can yield substantial health returns even in high-SDI settings (12, 29). The availability of advanced surgical techniques (30), multidisciplinary cardiac teams, and centralized high-volume pediatric cardiac programs further supports favorable outcomes even in anatomically complex cases. This reinforces the value of health system foresight and strategic reinvestment, which allow development to translate continuously into measurable health gains.

In the present study, forecasting models incorporating SDI projected higher CHA burden for Brunei Darussalam and Viet Nam than models based on YLL trends alone. This discrepancy reflects the nonlinear, inflection-point nature of YLL reduction in both countries—patterns that are poorly captured by the gradual and steady trajectory of SDI. In both cases, YLL improvements appear to be policy-driven and temporally decoupled from SDI growth. These trends suggest that significant reductions in CHA-related mortality were likely driven by targeted health system interventions rather than broad development progress alone. A report projected that Brunei will achieve a high overall SDG score of 90.0 by 2030, especially excelling in MCH and environmental health (25). Indeed, the variation can be attributed partly to stochastic variation, particularly given the small population of the country. However, the present study complemented the analysis with EAPC, which captures long-term temporal trends and reduces the influence of short-term random fluctuations (4), suggesting that the observed pattern reflects a genuine underlying trajectory.

In the present study, Viet Nam and Thailand were among the countries with close agreement between the trend-based and SDI-adjusted projections, suggesting that development gains have been relatively well translated into improvements in CHA care. However, in Viet Nam, the slight divergence observed—where trend-based projections are occasionally lower than SDI-adjusted estimates—was not consistent across the forecasted years. A previous study observed that while Viet Nam improved in resource allocation efficiency, it continued to lag in service delivery (12). This suggests that health outcomes, including CHA-related mortality, may have improved through targeted efforts rather than broad systemic progress. Similarly, Thailand's health ecosystem has been shaped over decades by its commitment to community-based care and Universal Health Coverage (UHC), which reached nearly 100% by 2015 (31, 32). This system is further supported by fiscal health policies, community health worker programs, and school-based prevention initiatives. As a result, Thailand is projected to score above 97.5 in UHC and near 100 in MCH and road safety indicators by 2030, making it one of ASEAN's strongest performers (25). These trajectories illustrate how SDI, in these contexts, acts as a true proxy for health system evolution, explaining why its integration improves YLL forecasts.

Countries with lower initial SDIs, such as Lao PDR, Cambodia, and Myanmar, demonstrated larger absolute YLL reductions but still rank high in forecasted YLL by 2030. For example, Lao PDR's YLL dropped from 2,528 in 2000 to 944 per 100,000 in 2021, yet projections show persistently high future burden. This may suggest a missed opportunity in aligning recent SDI growth with earlier scale-up of pediatric cardiac services (33–35). Similarly, Timor Leste, despite improvements, is projected to maintain high YLL levels unless more aggressive health interventions are pursued. The comparison of trend-based and SDI-adjusted forecasts revealed that adjusting for SDI generally narrows uncertainty and reflects more plausible trajectories in most countries. In nations like Cambodia and Lao PDR, the divergence between the two models indicates potential vulnerability if development gains do not translate into service expansion.

Our overall findings strengthen the argument that premature deaths from congenital heart anomalies are preventable. Forecasting results reveal a general downward trend in YLL across ASEAN countries, with sharper declines observed in nations that have either maintained high levels of sociodemographic development or translated improvements in development into health gains. The trend is similar to that observed in a previously reported study (26). The sharp contrasts between countries demonstrate that early adoption of congenital screening, newborn surgery access, and health financing can prevent avoidable mortality (26–28, 30). Strategically, countries should respond based on three priority tracks: (1) sustaining gains in high-performing systems by investing in innovation and specialist workforce; (2) accelerating early diagnosis and referral in mid-SDI countries through universal health coverage and task-shifting and (3) securing foundational access in low-SDI countries by integrating CHD care into maternal and child health platforms (36–39). Regional partnerships for pediatric cardiac surgery training and pooled procurement may also help close the gap.

It is noteworthy that although the GBD-based YLL data provide consistent estimates across countries and years, they rely on modeling assumptions and may not fully capture local diagnostic or reporting biases, especially in lower-SDI countries where underdiagnosis of CHAs is more likely. Additionally, the GBD cause hierarchy aggregates congenital cardiac conditions into a single category, preventing stratification by lesion complexity; therefore, the observed reductions in YLL may disproportionately reflect improvements in the management of simpler lesions, whereas outcomes for complex CHAs may remain substantially worse in settings with limited pediatric cardiac surgical capacity. This may result in underestimated YLL values in contexts with limited surveillance systems or low access to diagnostic facilities. Additionally, the forecasting analysis in the present study relies on a “model-on-model” framework. While this approach allows standardized cross-country comparisons (using GBD estimates) and long-term forecasting, it may compound uncertainty because errors or assumptions embedded in the original GBD models may propagate through the projection model. Consequently, the forecasts should be interpreted as scenario-based estimates rather than precise predictions. Further, the SDI projections used for ARIMAX forecasting are based on simple extrapolations of recent trends (linear or exponential depending on EAPC strata). These projections do not incorporate potential shocks (like pandemics or economic crises) or nonlinear policy shifts, which may alter the trajectory of development and healthcare access. Lastly, while we stratified forecasts by country, within-country disparities, such as rural–urban divides, geographic remoteness, or ethnic inequities, were not explored. This limits the granularity of our policy recommendations, particularly in large and diverse nations like Indonesia or the Philippines. Despite these limitations, our study provides valuable insight into the trajectory of preventable premature mortality due to CHAs and reinforces the need for strategic regional health planning and targeted congenital cardiac care improvements across ASEAN countries.

## 5 Conclusion

While several countries like Singapore, Malaysia, Thailand and Brunei Darussalam have successfully reduced premature mortality from CHAs through sustained, system-wide interventions, our findings indicate that such improvements are neither automatic nor uniform across ASEAN. Singapore's progress has been supported by an integrated congenital cardiac care system that includes routine prenatal detection, early neonatal screening, rapid referral pathways, and access to specialized high-volume pediatric cardiac surgical centers. Such coordinated systems allow timely intervention across the spectrum of congenital heart defects, including complex lesions, thereby contributing to sustained reductions in premature mortality. Meanwhile, Thailand's health system has been shaped by a long-standing commitment to UHC, supported by fiscal health policies, community health worker programs, and school-based prevention initiatives. Despite these regional success stories, several countries have shown limited gains in reducing CHA-attributable YLL, highlighting missed opportunities to align health investments with actual disease burden. Importantly, future surveillance systems should enable stratification by lesion complexity to better capture progress in managing both simple and complex congenital heart defects. Forecasting results further underscore that progress may stagnate or reverse without targeted action. Thus, reducing premature CHA mortality is feasible but not inevitable, where it depends on deliberate, burden-specific strategies, informed by evidence and committed policy leadership rather than passive reliance on sociodemographic progress.

## Data Availability

Publicly available datasets were analyzed in this study. This data can be found here: Global Burden of Disease (GBD) portal (http://ghdx.healthdata.org/gbd-results-tool).
